# Threshold-based quantification of fatty degeneration in the supraspinatus muscle on MRI as an alternative method to Goutallier classification and single-voxel MR spectroscopy

**DOI:** 10.1186/s12891-020-03400-4

**Published:** 2020-06-09

**Authors:** Dokwan Lee, Ki-Taek Hong, Wonhee Lee, Eun Kyung Khil, Guen Young Lee, Jung-Ah Choi, Yongnam Song

**Affiliations:** 1 Department of Mechanical Engineering, Korea University, Korea University Engineering Campus, Innovation Hall, Room 306, Anam-dong, Seongbuk-gu, Seoul, 02841 South Korea; 2 Department of Radiology, Hallym University College of Medicine, Hallym University Dongtan Sacred Heart Hospital, Hwaseong, Gyeonggi-do South Korea; 3 Department of Radiology, Chung-Ang University Hospital, Seoul, South Korea

**Keywords:** Rotator cuff, Fatty infiltration, Atrophy, Goutallier classification, Magnetic resonance imaging, MR spectroscopy

## Abstract

**Background:**

Conventional fat quantification methods for rotator cuff muscles have various limitations, such as inconsistent reliabilities of the Goutallier grades and need for advanced techniques in quantitative MRI sequences. We aimed to examine a threshold-based fat quantification method in the supraspinatus muscle on standard T1-weighted MR images and compare the threshold-based method with Goutallier grades and MR spectroscopy.

**Methods:**

We retrospectively examined 38 symptomatic patients, who underwent T1 and T2-weighted fast spin-echo MR imaging and a single voxel spin-echo MR spectroscopy. The supraspinatus muscle and fossa were manually segmented in T1-weighted sagittal images and clustering-based thresholding was applied to quantify the fat fractions in the segmented areas using custom MATLAB software. Threshold-based fat fractions were compared with the Goutallier grades and MR spectroscopy fat/water ratios. A one-way analysis of variance and Pearson correlation were tested in the MATLAB software.

**Results:**

Inter-observer reliability of threshold-based fat fractions for the supraspinatus muscle and fossa were 0.977 and 0.990 respectively, whereas the reliability of the Goutallier grading was 0.798. Threshold-based fat fractions in the supraspinatus fossa were significantly different between various Goutallier grades (one-way ANOVA, *p* < 0.001). Threshold-based fat fractions in the supraspinatus muscle strongly correlated with the MR spectroscopy fat/water ratio (Pearson correlation R-square = 0.83).

**Conclusions:**

Threshold-based fat quantification on standard T1-weighted MR images was highly reliable and produced comparable results to conventional Goutallier grades and MR spectroscopy fat/water ratios and could serve as an alternative method for accurate fat quantification in rotator cuff muscles.

## Background

Fatty infiltration and atrophy of the rotator cuff muscles have been thought to be secondary signs of chronic stages of muscle tears and pose a potential risk of re-tears after repair surgeries. Although some studies have reported delayed fatty degenerations after successful repairs, fatty infiltration and atrophy of the rotator cuff muscles have been known to be irreversible and may result in pathologic conditions of the glenohumeral joint, such as rotator cuff arthropathies [1–3]. Thus, surgical repairs of torn rotator cuff muscles must be considered prior to the progression of severe fatty infiltration or atrophy of the muscles [4–6]. Various computed tomography (CT) and magnetic resonance imaging (MRI) techniques have been introduced to non-invasively examine the degree of fatty degeneration of the rotator cuff muscles [7]. However, no method has been established as a gold-standard for the quantification of muscular fat fraction owing to the limitations in consistency and accessibility of the techniques in daily clinical applications.

The Goutallier classification has been the most popular method to evaluate the quality of rotator cuff muscles before surgery. The Goutallier method was originally designed as a semi-quantitative grading system of degenerative rotator cuff muscles with five different grades from grade 0 (no fat inside the muscle) to grade 4 (more than 50% fat inside the muscle) in CT images. Currently, a similar grading system is widely applied in MR images [8–10]. The Goutallier grading method is fast and convenient because the grades of muscle degenerations are visually evaluated on standard T1-weighted MR arthrographic images. Although the Goutallier grades have been reported to correlate with the severity of fatty infiltration in the rotator cuff muscles, their inconsistent intra-observer and inter-observer agreements need to be improved for use in accurate clinical diagnostic applications [11–14].

Recently, the single-voxel proton MR spectroscopy (MRS) was shown to produce accurate and reliable measurements of the fat content in the rotator cuff muscles by comparing the strength of the fat and water signals in the frequency domain of MR images [15, 16]. However, spectroscopic data in a limited single-voxel may not sufficiently describe the condition of an entire muscle. A new SPLASH technique expanded the single-voxel MRS method into a multi-voxel measurement, which collects magnetic signals of the fat and water molecules within the whole muscle cross-sections to accurately describe the degree of muscle degenerations in the entire muscle [17–19]. Multi-echo Dixon-based MRI was also actively investigated as an alternative method for the quantification of fat tissues inside the rotator cuff muscles. Dixon-based MRI measures the signal intensity values in separately acquired fat-only and water-only MR images [16, 20–22]. Fat fractions in Dixon-based MRI are determined by calculating the ratio of pixel intensity values between the fat-only and water-only images at all the image pixels.

Although MR spectroscopy and Dixon-based MRI have been studied as an accurate and reliable estimation of the fat fraction in muscle tissues by using the unique magnetic relaxation characteristics of the fat and water molecules, these techniques are not commonly used in daily clinical practice because substantial technical efforts are required for additional imaging procedures. Thus, a reliable and technically simple fat quantification method on standard T1-weighted MR images would allow an easy assessment of the degree of fatty degeneration in muscle tissues for small clinics with limited imaging resources. In this study, we aimed to quantify the fat content of the supraspinatus muscle on existing standard T1-weighted MR images by applying image thresholding techniques. Then, the threshold-based quantification results were compared with the conventional Goutallier classifications and MRS fat/water ratios to examine whether the new threshold-based approach is correlated with previously established methods. This new method may result in early diagnoses of muscle damages and improve surgical outcomes of repair surgeries.

## Methods

### Patients

This study was approved by the institutional review board, and informed consent was waived due to the retrospective nature of the study. We retrospectively examined the supraspinatus muscle on MR images of symptomatic patients from the outpatient shoulder clinic to evaluate the rotator cuff tendon status as well as muscle quality for preoperative evaluation. Total 48 patients were completed both proton MR spectroscopy and MR arthrography between January 2009 and December 2012. Patients with previous history of shoulder trauma (*n* = 3), surgery (*n* = 1), infection (*n* = 1), rheumatoid arthritis (*n* = 1), and congenital anomaly (*n* = 1) were excluded. MR images with artifacts from respiratory motion (*n* = 3) were also excluded. Finally, this study included 38 patients with 22 females (mean age: 63.05 ± 11.09 years, age range: 37–83 years) and 16 males (mean age: 53.07 ± 14.81 years, age range: 23–79 years). The ages of the female patients were significantly higher than those of the male patients (two sample t-test, *p* < 0.04). Supraspinatus pathology of the study population included normal (*n* = 4), tendinopathy (*n* = 6), articular-side low grade partial tears (*n* = 5), bursal-side high grade partial tears (*n* = 6), and full thickness tears (*n* = 17). A total of 16 patients eventually underwent rotator cuff repair surgeries. Inclusion criteria were patients with suspected rotator cuff pathology, possibly considering repair surgeries.

### MR imaging

**Fig. 1:**
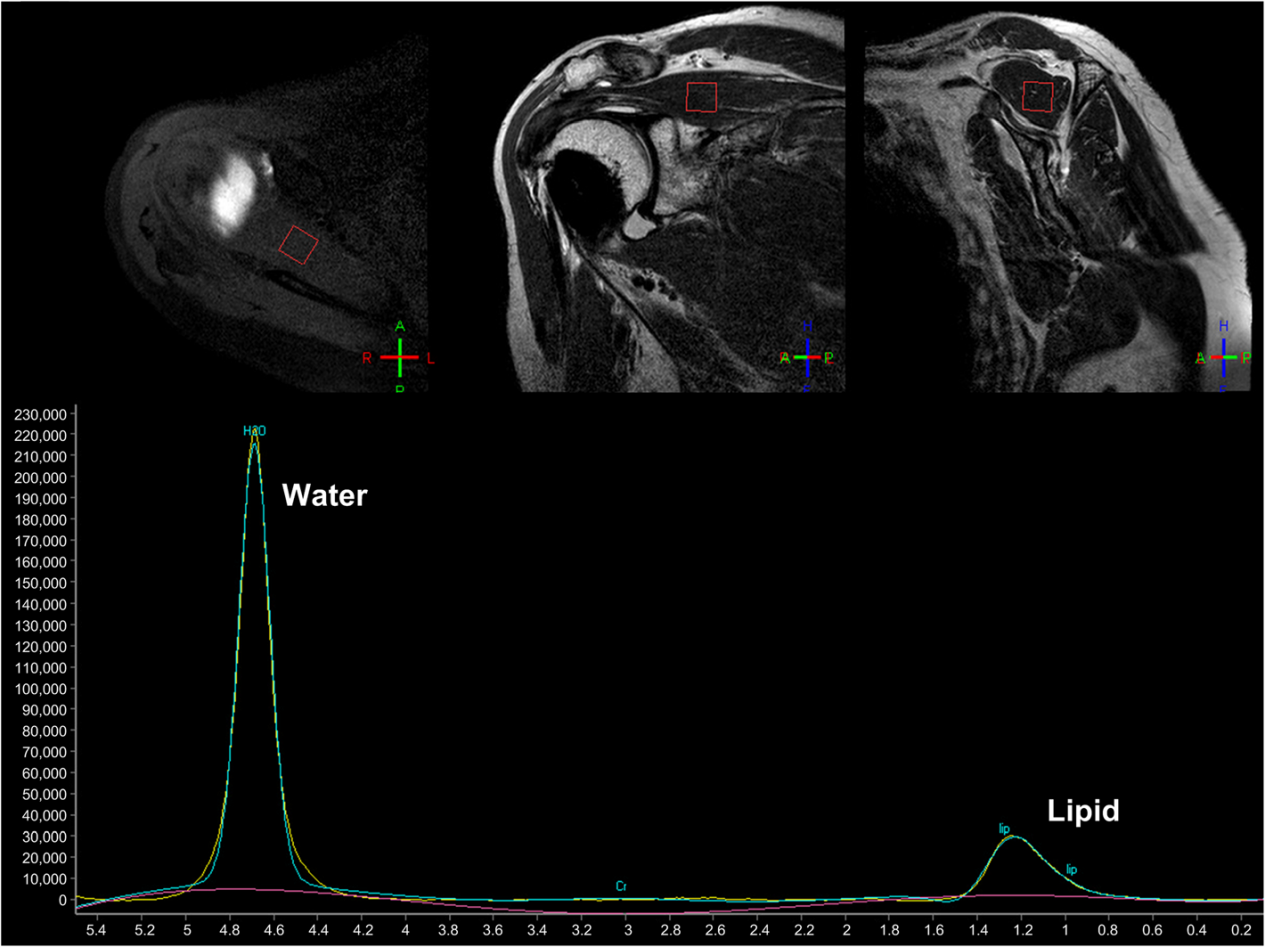
An example of the voxel location and spectroscopy measurement is presented

A 3 T MRI scanner (Achieva 3.0 T, Philips, Netherlands) with a flex-M shoulder array coil was used for the proton MR spectroscopy and MR arthrography. First, single voxel (10 × 10 × 10 mm^3^) spin-echo proton MR spectroscopy without water saturation (shimming procedure: volume shimming, number of excitation: 32, repetition time (TR): 4000 ms, echo time (TE): 35 ms, bandwidth (BW): 2000, scan time: 3 min 20 s) was acquired at the center of the supraspinatus muscle at the site of the largest muscle cross-section prior to MR arthrography. The location of the voxel was manually determined by navigating the oblique coronal and sagittal MR images of the shoulder. Then, a Gaussian fit of the water and lipid peaks was performed to calculate the magnitude and location of the signals by using SpectroView software (Philips Healthcare, Amsterdam, Netherlands) (Fig. 1). Frequencies of lipid and water peaks were around 1.3 ppm and 4.7 ppm respectively.

MR arthrography was obtained after intra-articular injection of 12 ml of gadodiamide (Omniscan, GE Healthcare, Princeton, NJ, USA) at a concentration of 2 mmol/L. Imaging protocol of MR arthrography was as follows: oblique coronal T1-weighted (TR: 500–1000 ms, TE: 20 ms, field of view (FOV): 140 × 140 mm^2^, matrix: 256 × 256, slice thickness: 2 mm, echo train length (ETL): 3–5) and T2-weighted fast spin-echo (FSE) sequence with and without fat suppression (TR: 3000–4500 ms, TE: 80–100 ms, FOV: 140 × 140 mm^2^, matrix: 256 × 256, slice thickness: 2 mm, ETL: 12–19), oblique sagittal T1-weighted (TR: 450–640 ms, TE: 8–11 ms, FOV: 140 × 140 mm^2^, matrix: 256 × 256, slice thickness: 2–3 mm, ETL: 6–8) and T2-weighted FSE sequence with fat suppression (TR: 3000–4500 ms, TE: 100 ms, FOV: 140 × 140 mm^2^, matrix: 256 × 256, slice thickness: 2 mm, ETL: 17), and transverse T1-weighted FSE images with fat suppression (TR: 440–600 ms, TE: 9–40 ms, FOV: 140 × 140 mm^2^, matrix: 256 × 256, slice thickness: 2 mm, ETL: 3–7).

### Goutallier classification

Multiple semi-quantitative MR-based Goutallier classifications were independently performed by three different raters. Examinations were done with oblique T1-weighted images at the location where the scapular spine meets the scapular neck and creates a “Y-shaped” bony configuration [23]. Personal information of MR images were removed prior to the Goutallier classification. Each rater completed their grading within a week. Each supraspinatus muscle was graded using the following five-point scale grading system: grade 0 (no fat inside the muscle), grade 1 (fatty streaks within muscle), grade 2 (evident fat, but fat less than 50% of muscle volume), grade 3 (approximately 50% fat), and grade 4 (more than 50% fat) [8, 24]. All the raters were musculoskeletal staff radiologists with experiences of more than five years in interpretation of musculoskeletal images. We did not perform a consensus training or a separated ‘tie-breaker’ consensus reading by a fourth musculoskeletal radiologist when the grades from the three raters did not agree. To reflect the majority opinion of the raters, the grades from each rater were averaged, and the averaged grades were then rounded to the nearest natural number as the final Goutallier grade of the muscle.

### Threshold-based quantification of fatty degeneration

Our threshold-based fat quantification method uses a clustering-based thresholding technique, which mathematically determines an optimal threshold value that separates the image pixels into foreground (bright) and background (dark) colors by analyzing the distribution of the pixel intensities within an image. We decided to use the Otsu thresholding [25, 26], which is one of the simplest clustering-based thresholding techniques, to examine the lower-bound efficacy of the threshold-based fat quantification idea. Briefly, the Otsu thresholding determines a threshold value at the pixel intensity, which maximizes the difference between the foreground (bright) and background (dark) pixels.

**Fig. 2:**
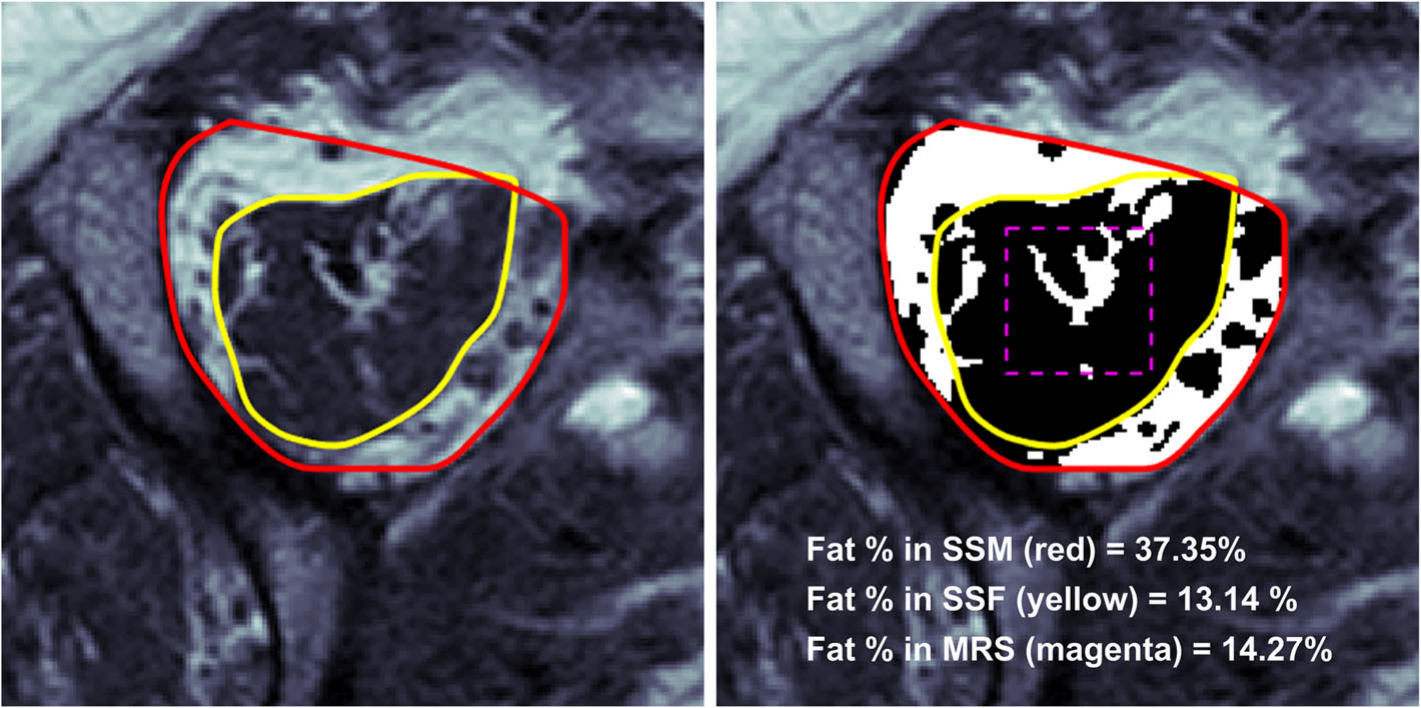
An example of threshold-based fat quantification is presented (SSM: supraspinatus muscle, SSF: supraspinatus fossa, MRS: 10 × 10 × 10 mm^3^ voxel for the MRS measurement)

The threshold-based fat quantification method was tested in DICOM images. We measured the threshold-based fat fractions in 1) the supraspinatus muscle and 2) the supraspinatus fossa which were manually segmented in T1-weighted oblique sagittal images using a custom MATLAB (R2017a, MathWorks, Massachusetts, USA) program (Fig. 2). Segmentations were performed in a total of three images, including the slide of MR spectroscopy and two more images before and after the spectroscopy slide. The fat fraction in an MR image was defined as the ratio between the number of foreground and background pixels within the segmented area. The fat fractions in the three consecutive images were averaged as the final fat content in the supraspinatus muscle of a patient. Segmentation and thresholding procedures were repeated by four independent observers to examine the inter-observer reliability. These observers consisted of three raters from the Goutallier classification and one additional researcher.

### Statistical analysis

A one-way analysis of variance (ANOVA) was performed to compare the differences between various Goutallier grade groups. Post hoc comparisons between each Goutallier group were performed using the Tukey honest significant difference test. The inter-observer reliability of the Goutallier grading and threshold-based fat quantification was evaluated by calculating the inter-class correlation coefficient (ICC) with 95% confident interval (CI). The Pearson correlation coefficient (R) and R-squared value were calculated to determine the relationship between the MRS fat/water ratios and threshold-based fat fraction measurements. A *p*-value less than 0.05 was considered to be a statistically significant difference. The statistical analysis was performed using the MATLAB software (R2017a, MathWorks, Massachusetts, USA).

## Results

### Reliability of Goutallier classification

The inter-observer reliability of the Goutallier grades between the three raters was fairly good with an ICC of 0.80 (95% CI: 0.67, 0.88). The distribution of the final Goutallier graded in our population is presented in Table 1.

**Table 1:** Summary of Goutallier grading results in the tested population

	Grade 0 (no fat)	Grade 1 (fatty streaks)	Grade 2 (fat < muscle)	Grade 3 (fat = muscle)	Grade 4 (fat > muscle)
Female	1	1	9	5	6
Male	2	5	8	0	1

### Reliability of threshold-based fat quantification

An example of threshold-based fat quantification is shown in Fig. 2. The inter-observer reliabilities of the threshold-based fat fraction measurement among four different observers were found to be excellent with ICCs of 0.98 (95% CI: 0.96, 0.99) and 0.99 (95% CI: 0.98, 0.99) for the supraspinatus muscle and supraspinatus fossa respectively. Coefficient of variations (the ratio of the standard deviation over the mean) in the segmented areas between difference observers were 3.56% (range: 0.72–9.27%) for the supraspinatus muscle and 8.59% (range: 2.35–22.79%) for the supraspinatus fossa.

### Relationship between threshold-based fat quantification and Goutallier classification

**Fig. 3:**
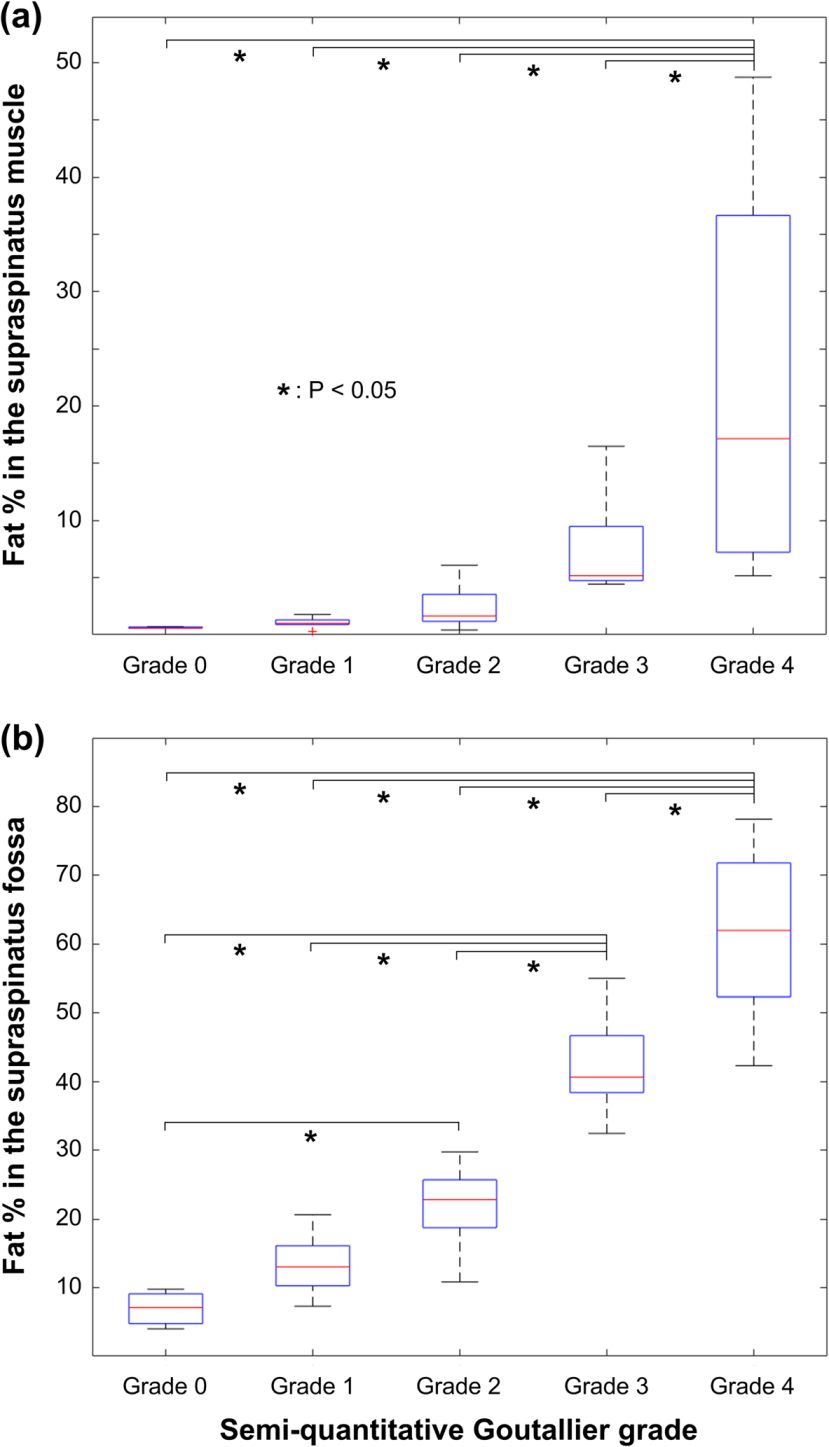
The threshold-based fat fractions in (**a**) the supraspinatus muscle and (**b**) the supraspinatus fossa in different Goutallier grades are presented (one-way ANOVA with post hoc Tukey honest significant difference test)

The mean fat fractions in the supraspinatus muscle and the supraspinatus fossa are listed in Table 2. These fat fraction values were calculated by using the segmentation result from one of 4 observers. The threshold-based fat fraction measurements in the supraspinatus muscle were not significantly different between various Goutallier grades (Tukey honest significant difference test, *p* > 0.6, Fig. 3) except for the grade 4 patients (*p* < 0.02). However, the threshold-based fat fractions in the supraspinatus fossa exhibited significant differences between most Goutallier grades (*p* < 0.03), except for the pairs of grade 0 and 1 (*p* > 0.7) and grade 1 and 2 (*p* > 0.1).

**Table 2:** Mean and 95% confidence interval (CI) of MRS fat/water ratio, threshold-based fat fraction (%) in the supraspinatus muscle, and threshold-based fat fraction (%) in the supraspinatus fossa for each Goutallier grade group

Mean (95% CI)	MRS fat/water ratio	Fat fraction (%) in the supraspinatus muscle	Fat fraction (%) in the supraspinatus fossa
Grade 0	0.03 (−0.03, 0.08)	0.62 (0.43, 0.81	6.96 (−0.23, 14.16)
Grade 1	0.03 (0.00, 0.06)	1.04 (0.52, 1.56)	13.40 (8.27, 18.53)
Grade 2	0.05 (0.03, 0.07)	2.36 (1.49, 3.24)	21.88 (19.08, 24.69)
Grade 3	0.26 (0.01, 0.51)	7.62 (1.34, 13.90)	42.47 (32.32, 52.62)
Grade 4	0.41 (0.04, 0.78)	22.29 (6.41, 38.18)	61.19 (49.27, 72.91)

### Relationship between threshold-based fat quantification and proton MR spectroscopy

**Fig. 4:**
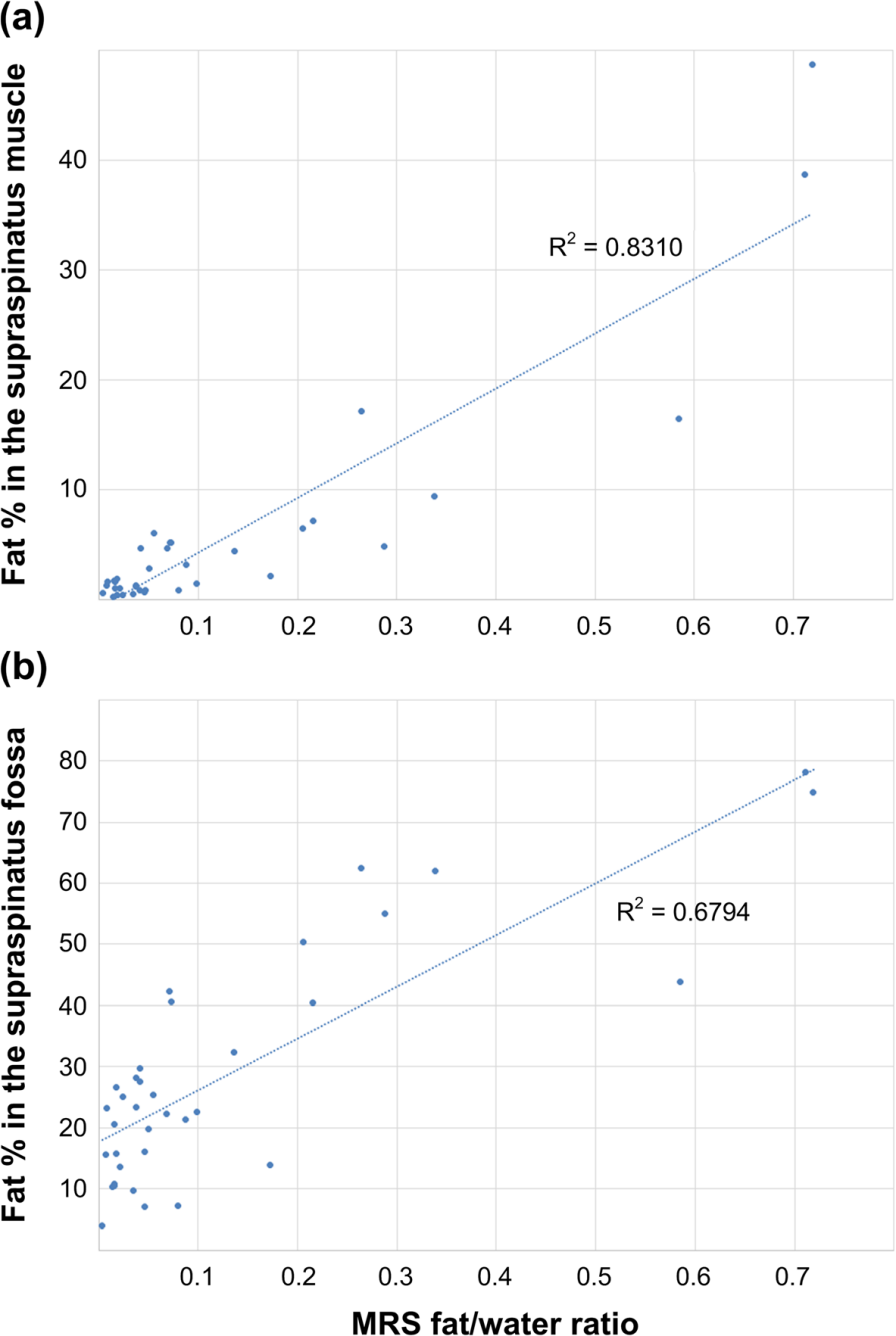
The fat/water ratio of MRS was compared with the threshold-based fat fraction measurements in (**a**) the supraspinatus muscle and (**b**) the region of the supraspinatus fossa (Pearson correlation test)

The fat/water ratio of MRS was correlated with the threshold-based fat fraction measurements in 1) the supraspinatus muscle and 2) the region of the supraspinatus fossa (Pearson correlation test, Fig. 4). Strong correlations were found between the MRS fat/water ratios and threshold-based fat fractions in the supraspinatus muscle (R-squared values = 0.83). However, the relationship between the fat/water ratios and the threshold-based fat fractions in the supraspinatus fossa were moderate (R-squared values = 0.68).

## Discussion

The semi-quantitative Goutallier grading system has been one of the most popular methods to examine the degree of fatty degeneration of the rotator cuff muscles due to the easy accessibility of standard T1-weighted MR images. However, the Goutallier grading method has questioned to be used as a clinical standard due to its inconsistent intra- and inter-observer reliabilities. Several studies have reported only ‘fair’ or ‘moderate’ intra- and inter-observer reliabilities in Goutallier grading system with the kappa values around 0.4–0.7 [11, 27, 28]. Reliabilities in Goutallier grades were also shown to be various with the type of fellowship training and the years in practice of observers [11]. Although recent MR spectroscopy and Dixon-based MRI techniques have been shown to improve the accuracy and consistency of fat fraction measurement, the use of these methods are limited because of the need for additional advanced techniques in MR imaging [29].

In this study, we quantified the fat fractions in the supraspinatus muscle and the supraspinatus fossa by using a threshold-based technique on standard T1-weighted MR images. Image thresholding significantly improved the reliability of the fat fraction measurements between different observers with inter-observer ICCs of over 0.97, which was much greater than the inter-observer ICC of 0.80 in the Goutallier grading system. Threshold-based fat fractions in the supraspinatus fossa were found to be significantly different between various Goutallier grades, and the fat fractions in the supraspinatus muscle were strongly correlated with the MRS fat/water ratio (R-squared values = 0.83). Our study resulted in slightly better reliabilities and correlation coefficients than the values shown in a recent study that examined fuzzy C-means thresholding technique in freely available MIPAV (Medical Image Processing, Analysis and Visualization, National Institutes of Health, Maryland, USA) software to estimate the degree of fatty infiltration in various shoulder muscles in 7 subjects, and found excellent inter-observer reliabilities (ICC = 0.95) and correlations (R-squared values = 0.7225) with the fat fractions by six-point Dixon MRI [29]. These results suggest that the threshold-based fat quantification method on standard T1-weighted MR images was highly reliable and produced comparable results to conventional Goutallier grades and MRS fat/water ratio without the need for additional MR techniques.

Inter-observer variations in the manual segmentation were found to be approximately 4–9% of the mean segmented areas between different observers in supraspinatus muscle and fossa. Simple boundary shapes of supraspinatus muscle and fossa with circular and rounded triangular profiles might reduce the disagreement of segmentation lines between difference observers. Our intra-observer variations in the segmented areas (4–9%) would alter the final fat content by approximately 3–8% for a muscle with an 80% fat fraction. However, these changes in the fat content would be smaller in the muscles with lower fat fractions. Because most of our patients exhibited the fat contents much lower than 80%, the effect of segmentation errors on the final fat content estimations would be minimal.

Differences in the threshold-based fat fraction of the supraspinatus fossa were found to be significant between different Goutallier grades, whereas the differences in the supraspinatus muscle fat content were minimal. Although the Goutallier grading system was designed to access the degree of fatty infiltration inside the rotator cuff muscles, our raters may consider a higher Goutallier grade for a patient with severe muscle atrophy because atrophy of the supraspinatus muscle is also believed to be an important sign of muscle degeneration. Our fat fraction measurements in the supraspinatus fossa would be similar to the conventional occupation ratio method which measures 2 dimensional areal ratios between the supraspinatus muscle and the supraspinatus fossa [7, 30]. The occupation ratio was reported to correlate with the degree of muscle degenerations which exhibited significantly smaller occupation ratios in shoulders with full-thickness massive supraspinatus muscle tears than the values in shoulders with less severe tears [30]. However, this occupation ratio is also unable to estimate internal fatty degenerations in the supraspinatus muscle. Although the relationship between the degree of muscle atrophy and degenerative muscle tears remains unclear [23], our study may suggest that the fat fractions in the supraspinatus fossa could also be considered as an effective alternative to the conventional Goutallier grading system since the total fat content in the supraspinatus fossa area reflects both fatty infiltration and atrophy of the supraspinatus muscle.

The threshold-based fat fractions in the supraspinatus muscle exhibited a strong correlation with the MRS fat/water ratio (Pearson correlation test, R-square = 0.83). MR spectroscopy measures magnetic signals from various molecules in muscle tissues. Thus, the relative strength of the fat signal over the signals from other tissues is believed to provide an accurate proportion of the fat tissue inside the entire muscular structure. However, the relationship between the MRS fat/water ratios and the threshold-based fat fractions in the supraspinatus fossa were moderate because the MR spectroscopy only measured intramuscular fats within a single voxel of the supraspinatus muscle. A strong correlation between the threshold-based fat contents and MRS fat/water ratios suggests that majority of fat tissues in the supraspinatus muscle could be visually recognized by image thresholding techniques in standard T1-weighted images. Our study showed that the clustering-based thresholding technique could accurately estimate internal fat content of the supraspinatus muscle with high reliabilities without an aid of image post-processing software.

2 dimensional assessments of muscular fatty degeneration in a single MR slide have been shown to inaccurately describe the true condition of an entire muscle. Because the fatty infiltration has been reported to be non-uniform throughout the muscle belly and the location of the single MR slide could be changed by muscle stretches after repair surgeries, 3 dimensional assessments of a whole muscle have been believed to significantly improve diagnostic outcomes. Unfortunately, current Goutallier classification method does not have established grading guidelines for multi-slide 3D images. A potential application of 3D Goutallier classification may require unacceptable amount of examination time for clinical applications. With the improvements of current automatic segmentation techniques, our threshold-based fat quantification method could be developed as a tool for the measurement of 3D fat content in a whole muscle to markedly improve the accuracy and reliability of clinical diagnosis on muscle degenerations.

This study has some limitations that need to be addressed. First, the Otsu thresholding used in this study is one of the simplest clustering-based thresholding methods. However, many modern advanced clustering-based thresholding techniques share the concept of the Otsu thresholding. Clustering-based thresholding methods are widely used in various medical image post-processing software and look promising for the quantification of fat content in the rotator cuff muscles because these techniques effectively remove the observer’s personal perceptions of the fat and muscle tissues in medical images. This study presented a lower-bound efficacy of the threshold-based fat quantification by using the simplest Otsu thresholding. The accuracy and reliability of the threshold-based fat quantifications can be improved by using advanced clustering-based thresholding algorithms. Second, MR spectroscopy was only measured in a single voxel of 10 × 10 × 10 mm^3^ at the central portion of the supraspinatus muscle. Our spectroscopy measurements only provided limited information of the muscle composition within the small voxel, which may not reflect the condition of the whole muscle. Unfortunately, we were unable to apply a multi-voxel SPLASH-type sequence [17–19] due to technical limitations. The 10 × 10 × 10 mm^3^ voxel of the spectroscopy was calculated to cover approximately 30% of the supraspinatus muscle cross-section. When we measured the relationship between the MRS fat/water ratio and the threshold-based fat content within the central 10 × 10 × 10 mm^3^ region, the correlation coefficient in the central voxel was found to be 0.88 (Pearson correlation test, R-square = 0.78), which was comparable to the correlation coefficient measured in the entire muscle area. Finally, our patient population was not uniform across different Goutallier grades. We had few cases of Goutallier grade 0 because only symptomatic patients considering rotator cuff repair surgery were included. Our irregular patient population did not produce enough cases for individual examinations of the threshold-based fat fractions between patient subgroups of different Goutallier grades or pathologies. Correlations between threshold-based fat quantification and proton MR spectroscopy (Fig. 4) could also be affected by the non-uniform patient distribution. Although a sufficient number of patients with healthy supraspinatus muscles (grade 0) would provide better results in statistical analyses, we were unable to scan additional non-symptomatic normal subjects for ethical issues. However, we believed that our results would be still useful for clinically relevant symptomatic patients with severe muscle degenerations. Moreover, separate power analyses revealed that the powers of ANOVA tests between difference Goutallier grades (Fig. 3) were over 0.95 and the powers of Pearson correlation tests (Fig. 4) were over 0.88 indicating small probabilities of type 2 errors.

The threshold-based fat quantification method is a standard image post-processing technique and easily employed in desktop-level computers. This threshold-based method should be a powerful tool for early and reliable diagnosis of muscle degenerations in small hospitals that are unable to perform quantitative MR imaging sequences such as MRS and Dixon-based MRI. Though further research on the threshold-based fat quantification needs to be followed, future advances in the thresholding technology and machine learning algorithms would continuously improve the accuracy of threshold-based fat measurements and may eventually become a potential alternative of Goutallier grading system and quantitative MR imaging technologies.

## Conclusions

In this study, we applied a clustering-based image thresholding technique on existing standard T1-weighted MR images to quantify the degree of fatty degeneration of the supraspinatus muscle. The threshold-based fat fraction measurements exhibited excellent reliabilities among different observers, and found strong correlations 1) between the fat fractions in the supraspinatus fossa and Goutallier grades, and 2) between the fat fractions in the supraspinatus muscle and MRS fat/water ratios.

## Data Availability

The datasets used and/or analyzed during the current study are available from the corresponding author on reasonable request.
